# Enalapril stimulates collagen biosynthesis through prolidase-dependent mechanism in cultured fibroblasts

**DOI:** 10.1007/s00210-015-1114-5

**Published:** 2015-03-17

**Authors:** Lukasz Szoka, Ewa Karna, Renata Pawlak Morka, Jerzy A. Palka

**Affiliations:** Department of Medicinal Chemistry, Medical University in Bialystok, Mickiewicza 2 D, 15-222 Bialystok, Poland

**Keywords:** Enalapril, Enalaprilat, Collagen biosynthesis, Fibroblasts, Prolidase

## Abstract

The mechanism of a lower incidence of dermatological manifestations in patients treated with enalapril compared to patients treated with other ACE-inhibitors, e.g., captopril, is not known. The finding that prolidase plays an important role in collagen biosynthesis and that some angiotensin-converting enzyme inhibitors affect prolidase activity led us to evaluate its effect on collagen biosynthesis in cultured human skin fibroblasts. Since insulin-like growth factor (IGF-I) and transforming growth factor beta 1 (TGF-β1) are the most potent stimulators of both collagen biosynthesis and prolidase activity, and prolidase is regulated by β_1_ integrin signaling, the effect of enalapril and enalaprilat on IGF-IR, TGF-β1, and β_1_ integrin receptor expressions was evaluated. Cells were treated with milimolar concentrations (0.3 and 0.5 mM) of enalapril and enalaprilat for 24 h. The activity of prolidase was determined by colorimetic assay. Collagen biosynthesis was evaluated by radiometric assay. Expression of signaling proteins was evaluated using Western blot. It was found that enalapril- and enalaprilat-dependent increase in prolidase activity and expression was accompanied by parallel increase in collagen biosynthesis. The exposure of the cells to 0.5 mM enalapril and enalaprilat contributed to increase in IGF-IR and α_2_β_1_ integrin receptor as well as TGF-β1 and NF-*κ*B p65 expressions. Enalapril- and enalaprilat-dependent increase of collagen biosynthesis in fibroblasts results from increase of prolidase activity and expression, which may undergo through activation of α_2_β_1_ integrin and IGF-IR signaling as well as upregulation of TGF-β1 and NF-*κ*B p65, the inhibitor of collagen gene expression.

## Introduction

Enalapril, (S)-1-[N-(1-etoxycarbonyl)-3-phenylpropyl]-L-proline, a prodrug, is converted by deesterification to angiotensin-converting enzyme inhibitor (ACE-I), enalaprilat, (S)-1-[N-(1-carboxy-3-phenylpropyl)-L-alanyl]-L-proline dihydrate, commonly used to control hypertension (Ovchinnikov et al. 2009). It was the first dicarboxylate-containing ACE inhibitor, which was developed to overcome the limitations of captopril. The sulfhydryl moiety was replaced by a carboxylate moiety, but additional modifications were required in its structure to achieve a similar potency to captopril.

It was documented that treatment of hypertensive patients with enalaprilat gives a lower incidence of dermatological manifestations than results from other ACE-I treatment—captopril (Davies et al. 1984). Structurally, enalaprilat is a dipeptide having L-proline as the C-terminal residue. Dipeptides of the x-pro type are substrates exclusively for prolidase. In contrast to captopril, enalaprilat had no inhibitory effect on porcine kidney prolidase (PKP) (King et al. 1989). This dipeptidase is present in all tissues and plays an important role in the recycling of proline from imidodipeptides (derived from degradation products of collagen) for collagen re-synthesis (Yaron and Naider 1993) and cell growth (Emmerson and Phang 1993). The efficiency of recycling of proline was found to be about 90 % (Jackson et al. 1975). It is evident that an absence of prolidase severely impedes the recycling of collagen proline. Some clinical symptoms related to collagen deficit can be attributed to prolidase deficiency (Freij et al. 1984; Cabrera et al. 2004; Lupi et al. 2008). On the other hand, an increased activity of liver prolidase was found during the fibrotic process (Myara et al. 1987). There was a positive correlation between prolidase activity and fibrosis scores in the lung (Türkbeyler et al. 2012). It suggests that the enzyme activity (despite the collagen gene expression) may be a step-limiting factor in regulation of collagen biosynthesis (Surazynski et al. 2008a). A significant increase in serum prolidase activity was observed in patients with hypertension, which was interpreted as evidence of increased collagen degradation with a higher collagen turnover rate in hypertension tissues, contributing to left ventricular hypertrophy (Demirbag et al. 2007).

Collagen, which accounts for about one third of total body proteins, is not only essential for the maintenance of connective tissue architecture. The interaction between cells and extracellular matrix (ECM) proteins, e.g., collagen, can regulate cellular gene expression, differentiation, and growth (Bissel 1981; Carey 1991). The interaction is mediated by specific cell surface receptors of integrin family. The α_2_β_1_ integrin is known as a main collagen receptor. Activation of this receptor by collagen ligation initiates cascade of signaling pathway including FAK, Src, Shc, Grb2, Sos, Ras, Raf and MAP kinases, ERK1, and ERK2 (Boudreau and Jones 1999). Decrease in collagen availability for integrin receptor interaction may therefore potentially alter cellular metabolism. Prolidase activity is stimulated through a signal mediated by collagen–β_1_ integrin receptor interaction (Palka and Phang 1997, 1998). This pathway is known to be involved in phosphorylation of several intracellular proteins, including prolidase (Surazynski et al. 2001).

Another important point of collagen biosynthesis regulation is at the level of insulin-like growth factor-I receptor (IGF-IR). IGF-I is one of the potent collagen-stimulating factor in collagen-synthesizing cells (Goldstein et al. 1989). Stimulated IGF-I receptor induces interaction of several signaling proteins, such as Grb2, Src, and Shc. This interaction allows activating further cascade of signaling pathway through Sos, Ras, and Raf proteins and, subsequently, two MAP kinases—ERK_1_ and ERK_2_ (Werner and Le Roith 2000). The end point of this phenomenon is induction of some transcription factors that regulate cellular metabolism. Some of these activities are regulated through NF-*κ*B, the known inhibitor of collagen gene expression (Kouba et al. 1999). On the other hand, transforming growth factor beta 1 (TGF-β1) may take part in the stimulation of collagen biosynthesis (Surazynski et al. 2010). It was documented in studies where anti-TGF-β1 antibody or gene silencing by si-TGF-β1 counteracted TGF-β1-dependent increase in collagen type I production (Jiang et al. 2012).

Considering the above mentioned factors, we studied the cellular mechanisms for the effect of enalapril-prodrug and enalaprilat-active form of the drug on collagen biosynthesis in cultured human dermal fibroblasts.

## Materials and methods

Alkaline phosphatase-labeled anti-mouse IgG, anti-rabbit IgG, and anti-goat IgG antibodies, bacterial collagenase, enalapril maleate salt and enalaprilat dihydrate (dissolved in DMEM), Fast BCIP/NBT reagent, L-glycyl-proline, L-proline, monoclonal (rabbit) TGF-β1 antibody, and monoclonal (mouse) anti-IGF-IR antibody were provided by Sigma Corp., USA., as were most other chemicals and buffers used. Dulbecco’s minimal essential medium (DMEM) and fetal bovine serum (FBS) used in cell culture were products of Gibco, USA. Glutamine, penicillin, and streptomycin were obtained from Quality Biologicals Inc., USA. Nitrocellulose membrane (0.2 μm), sodium dodecylsulphate (SDS), polyacrylamide, molecular weight standards, and Coomassie Briliant Blue R-250 were received from Bio-Rad Laboratories, USA. L-5[^3^H] proline (28 Ci/mmol) was purchased from Amersham, UK. Monoclonal (mouse) anti-β_1_, polyclonal (rabbit) anti-α_2_-integrin and NF-*κ*B p65 antibodies, and polyclonal (goat) anti-β-actin antibody were the products of Santa Cruz Biotechnology Inc., USA. Polyclonal anti-human prolidase antibody was donated by Dr. James Phang (NCI-Frederick Cancer Research and Development Center, Frederick, MD, USA).

### Tissue culture

All studies were performed on normal human skin fibroblasts (CRL-1474), which were purchased from American Type Culture Collection, Manassas, VA, USA. The cells were maintained in DMEM supplemented with 10 % fetal bovine serum (FBS), 2 mmol/l glutamine, 50 U/ml penicillin, and 50 μg/ml streptomycin at 37 °C in a 5 % CO_2_ incubator. Cells were counted in hemocytometer and cultured at 1 × 10^5^ cells per well in 2 ml of growth medium in six-well plates (Costar). Cells reached confluence at day 6, and in most cases, such cells were used for assays. Cells were used in the 8th to 14th passages.

### Determination of prolidase activity

The activity of prolidase was determined according to the method of Myara et al. (1982). Protein concentration was measured by the method of Lowry et al. (1951). Enzyme activity was reported as nanomoles of proline released from synthetic substrate, during one minute per milligram of supernatant protein of cell homogenate.

### Collagen production

Incorporation of radioactive precursor into proteins was measured after labeling of confluent cells in growth medium with enalapril-prodrug and enalaprilat-active form for 24 h with 5[^3^H] proline (5 μCi/ml, 28 Ci/mM) as described previously (Oyamada et al. 1990). Incorporation of tracer into collagen was determined by digesting proteins with purified *Clostridium histolyticum* collagenase, according to the method of Peterkofsky et al. (1982). Results are shown as combined values for cell plus medium fractions.

### SDS-PAGE

Slab SDS-polyacrylamide gel electrophoresis (PAGE) was used, according to the method of Laemmli (1970), by using 10 % SDS-polyacrylamide gel.

### Western immunoblot analysis

After SDS-PAGE, the gels were allowed to equilibrate for 5 min in 25 mmol/l Tris and 0.2 mol/l glycine in 20 % (*v*/*v*) methanol. The protein was transferred to 0.2-μm pore-sized nitrocellulose at 100 mA for 1 h by using a LKB 2117 Multiphor II electrophoresis unit. The nitrocellulose was incubated with the following: monoclonal anti-β_1_, polyclonal anti-α_2_-integrin, and NF-*κ*B p65 antibodies at concentration 1:1000; polyclonal antibody against β-actin at concentration 1:3000; polyclonal antibody against prolidase at concentration 1:5000; and monoclonal antibodies against IGF-IR and TGF-β1 at concentration 1:1000 in 5 % dried milk in TBS-T (20 mmol/l Tris-HCl buffer, pH 7.4, containing 150 mmol/l NaCl and 0.05 % Tween 20) for 1 h. In order to analyze β_1_ integrin subunit and IGF-IR second antibody-alkaline phosphatase conjugated, anti-mouse IgG (whole molecule) was added at concentration 1:7500 in TBS-T; in order to analyze prolidase, α_2_ integrin subunit, TGF-β1, and NF-*κ*B p65 second antibody alkaline phosphatase conjugated, anti-rabbit IgG (whole molecule) was added at concentration 1:5000; and, in order to analyze β-actin second antibody-alkaline phosphatase conjugated, anti-goat IgG (whole molecule) was added at concentration 1:5000 in TBS-T and incubated for 30 min with slow shaking. Then, nitrocellulose was washed with TBS-T (5 × 5 min) and submitted to Sigma-Fast BCIP/NBT reagent. The intensity of the bands was quantified by densitometric analysis.

### Statistics

In all experiments, the mean values for three independent experiments done in duplicates ± standard deviation (SD) were calculated. The results were submitted to statistical analysis using one- way ANOVA followed by Tukey test, accepting *P < 0.05 as significant versus control.

## Results

**Fig. 1:**
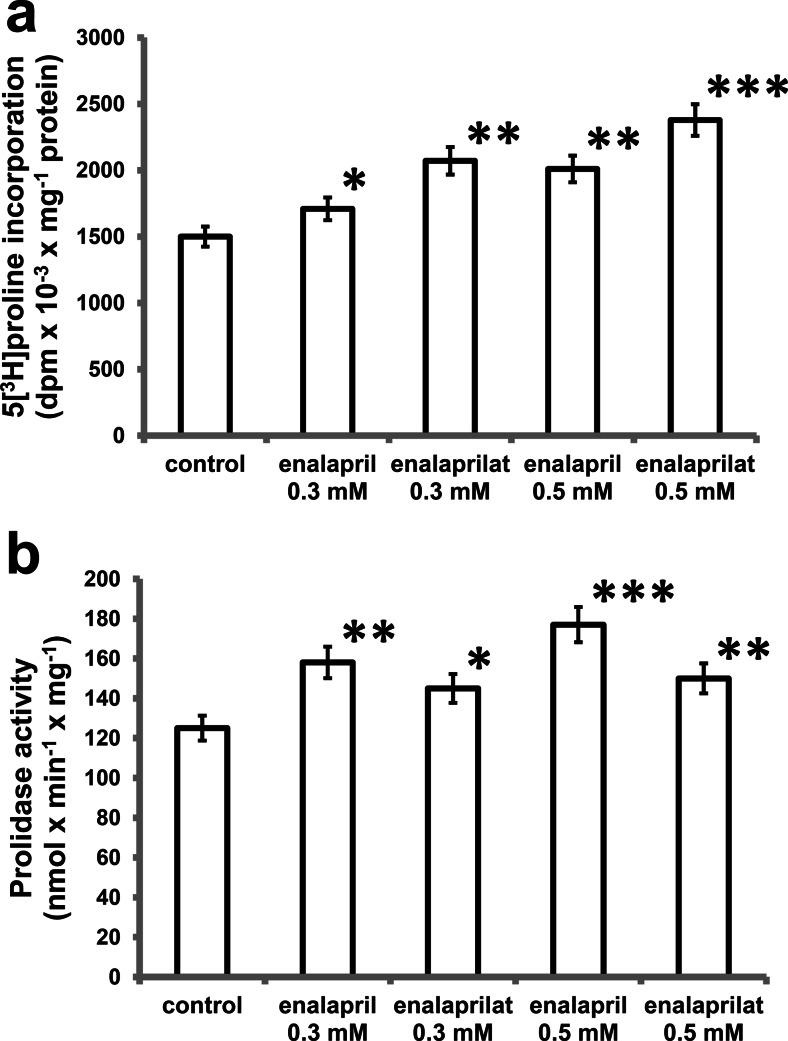
Collagen biosynthesis (**a**) measured as 5[^3^H] proline incorporation into proteins susceptible to the action of bacterial collagenase and prolidase activity (**b**) in confluent human skin fibroblasts incubated for 24 h in the medium containing 10 % FBS and different concentrations of enalapril and enalaprilat. The results present the mean values from six assays ± SD. *P < 0.05; **P < 0.01; ***P < 0.001 compared with the control

**Fig. 2:**
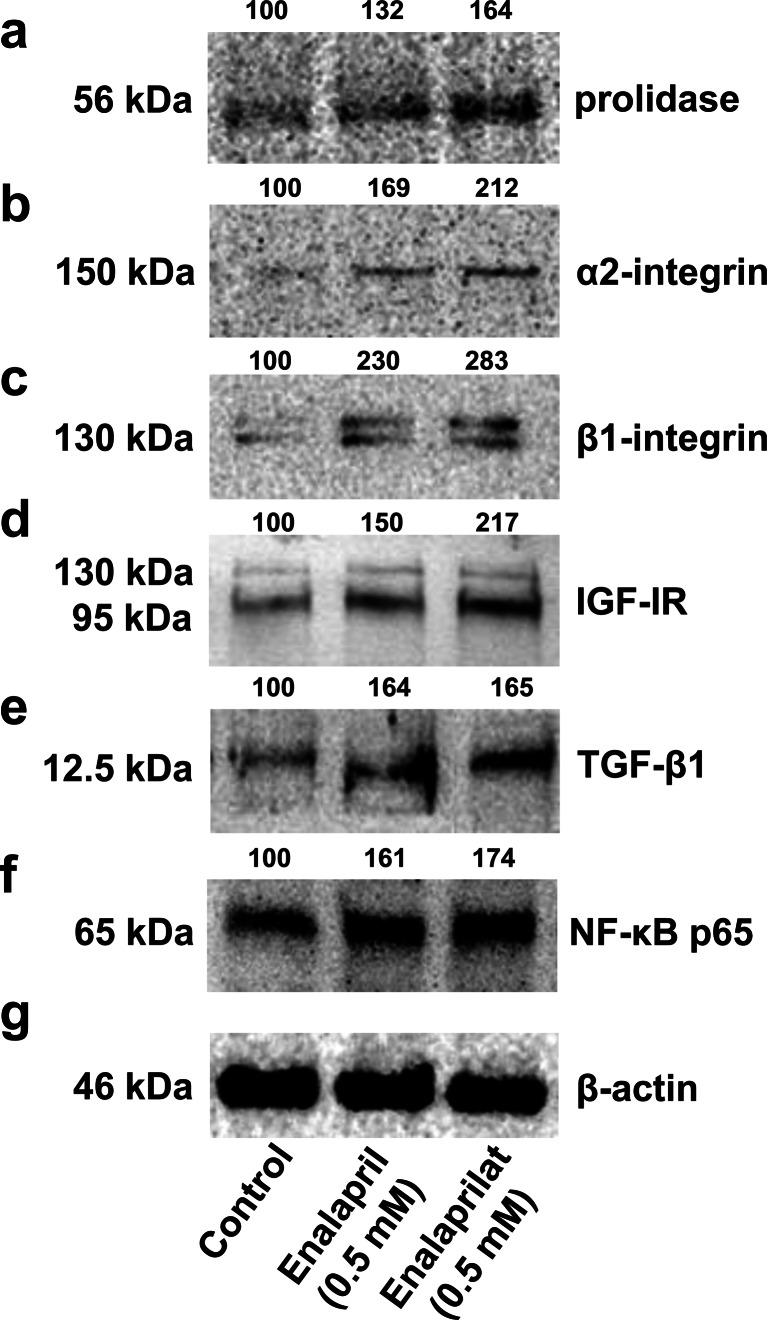
Western blot analysis for prolidase (**a**), α_2_ integrin receptor (**b**), β_1_ integrin receptor (**c**), IGF receptor (**d**), TGF-β1 (**e**), and NF-κB p65 (**f**) in control human skin fibroblasts (*lane 1*) and cultured in the medium containing 0.5 mM of enalapril (*lane 2*) or 0.5 mM of enalaprilat (*lane 3*). The mean values of six pooled cell homogenate extracts from six separate experiments are presented. The intensity of the bands was quantified by densitometric analysis. Densitometry was done with BioSpectrum Imaging System and presented as an arbitrary units. The same amount of supernatant protein (20 μg) was run in each lane. The expression of *β*-actin served as a control for protein loading (**g**)

Collagen biosynthesis and prolidase activity were measured in confluent human dermal fibroblasts, which have been treated with 0.3 and 0.5 mM enalapril and enalaprilat (dissolved in DMEM). As can be seen in Fig. 2, 24-h incubation of confluent fibroblasts in the medium containing 10 % of FBS and different concentrations of enalapril or enalaprilat contributed to increase in collagen biosynthesis (Fig. 1a) and prolidase activity (Fig. 1b) in a dose-dependent manner. At 0.3 and 0.5 mM, enalapril induced increase in collagen biosynthesis to about 114 and 134 %, respectively. After 24-h incubation, enalaprilat at 0.3 and 0.5 mM contributed also to increase in collagen biosynthesis to about 138 and 159 %, respectively (Fig. 1a).

Prolidase activity was increased to about 126 and 132 % of control at 0.3 and 0.5 mM of enalapril, respectively. At 0.3 and 0.5 mM, enalaprilat induced prolidase activity to about 128 and 142 % of control, respectively (Fig. 1b).

Increase in prolidase activity due to 24-h treatment of fibroblasts with enalapril or enalaprilat was accompanied by increase in the expression of the enzyme as shown by Western immunoblot analysis (Fig. 2a).

The data shows that both, enalapril and enalaprilat, induced increase in collagen biosynthesis in skin fibroblasts and suggests that the increase may result from activation of prolidase activity and expression.

Collagen biosynthesis and prolidase activity were previously shown to be regulated due to the signal induced by activated α_2_β_1_ integrin receptor (Palka and Phang 1997; Ivaska et al. 1999) as well as insulin-like growth factor-I receptor (IGF-IR) (Goldstein et al. 1989) and transforming growth factor beta (TGF beta) (McAnulty et al. 1991; Surazynski et al. 2010). Therefore, the expression of α_2_β_1_ integrin receptor (receptor for type I collagen), IGF-IR, and TGF-β1 were measured by Western immunoblot analysis. As can be seen in Fig. 2b, c, 24-h treatment of fibroblasts with 0.5 mM enalapril or enalaprilat contributed to a distinct increase in the expression of α_2_ and β_1_ integrin subunits, compared to the control cells (Fig. 2b, c, line 1). In addition, as shown in Fig. 2d, an increase in IGF-I receptor expression was found in enalapril- and enalaprilat-treated cells, compared to control cells. Simultaneously, we have found an increase in the expression of TGF-β1 (Fig. 2e) and NF-*κ*B p65 (Fig. 2f), the known inhibitor of collagen gene expression (Kouba et al. 1999), compared to control cells (Fig. 2e, f, line 1).

In view of this data, it seems that ability of enalapril and enalaprilat to induce collagen biosynthesis may involve increase in prolidase activity and expression, and increase in α_2_β_1_ integrin, IGF-I receptor, TGF-β1, and NF-*κ*B p65 expressions.

## Discussion

The finding that enalaprilat gives a lower incidence of dermatological manifestations than results from other ACE-I treatment, captopril (Davies et al. 1984), led us to investigate its role in collagen biosynthesis in fibroblasts—the main collagen synthesizing cells (Makela et al. 1990). It is well established that prolidase, providing proline for collagen biosynthesis, is a rate-limiting factor in regulation of this process (Karna et al. 2000, 2001; Galicka et al. 2001; Surazynski et al. 2008a). Therefore, the mechanism of prolidase activity regulation is of considerable interest. In contrast to captopril, enalaprilat had no inhibitory effect on porcine kidney prolidase (PKP) (King et al. 1989). In fact, in our previous studies (Karna et al. 2010), we found that exposure of fibroblasts to captopril contributed to a decrease in prolidase activity, and expression of α_2_β_1_ integrin receptor and IGF-IR, suggesting underlying mechanism of captopril-dependent inhibition of collagen biosynthesis. It seems that in view of dermatological manifestation that accompanies captopril therapy, the above mechanism may be of great importance.

In the present study, we found that enalapril and enalaprilat contributed to increase in prolidase activity. Their effect on the activity of other enzymes was also observed (Männistö et al. 2001). The constellation of changes induced by studied ACE inhibitors found in this study suggests an important role of prolidase in upregulation of collagen synthesis. It supports our previous studies on prolidase-dependent regulation of collagen biosynthesis (Karna et al. 2006). Several other studies suggest that prolidase-dependent regulation of collagen biosynthesis may take place at the transcriptional level. The transfection of colorectal cancer cells with prolidase vector inhibited NF-κB expression (Surazynski et al. 2008a), well-recognized inhibitor of expression of α1 and α2 subunits of type I collagen (Kouba et al. 1999; Rippe et al. 1999; Miltyk et al. 2007). Another evidence for the role of prolidase in regulation of NF-κB expression provides experiment showing that inhibition of prolidase activity by Cbz-Pro contributed to upregulation of NF-κB expression in fibroblasts (Surazynski et al. 2008a). In contrast, our data showed that enalapril- and enalaprilat-dependent increase in collagen biosynthesis is accompanied by an increase in the expression of NF-κB p65. Although it is accepted that enalapril inhibits NF-kB-induced inflammatory responses in several experimental “in vivo” models (Kushwaha and Jena 2012), our data on enalapril effect on NF-kB p65 expression in cultured fibroblasts showed contrary results. It cannot be ruled out that in the experimental conditions, enalapril upregulates coordinately expression of both NF-kB and IGF-IR (shown in our studies) since IGF-IR promoter contains NF-kB binding site (Ma et al. 2006). Such a hypothesis seems to be reasonable since recently it was found that enalapril and nifedipine differentially regulate 33 genes involved in the pathogenesis of cardiovascular diseases (Lee et al. 2013).

Enalaprilat as an angiotensin-converting enzyme inhibitor (ACE-I) has a pseudo-peptide structure with a proline residue at the position P′2. This proline residue is also present in the natural substrate of ACE, angiotensin I, and it has been suggested that the preference of proline at position P′2 of the peptide may reside in its rigid conformation that allows the carboxy terminus to be placed in a favorable alignment for the interaction with a positively charged amino acid of the active site of the enzyme. Important role of proline moiety at the binding process was proposed (Andújar-Sánchez et al. 2004).

There are some evidence that ACE-Is increase synthesis of collagen, one of the major constituents of the atherosclerotic cap (Claridge et al. 2004). Rhaleb et al. (2001) found that ACE-Is through indirect mechanism may contribute to regulation of collagen synthesis. We used a range of ACE inhibitors at concentrations up to 0.5 mM because of our previous experience with captopril (Karna et al. 2010). Some studies “in vitro” were performed with higher ACE-I concentration (10 mmol/l) (Mailloux et al. 2003).

The use of ACE-Is in other animal models of atherosclerosis has shown an increase in the extracellular matrix proteins (Rabbani and Topol 1999). These phenomena were explained by the upregulation of collagen synthesis, and the presence of increased quantities of collagen in the fibrous cap (Claridge et al. 2004). Moreover, angiotensinogen, angiotensin-converting enzyme (ACE), and angiotensin II receptor are expressed in skin (Steckelings et al. 2004). It is known that ACE inhibitors might suppress skin disfunction and inflammatory response mediated through the angiotensin II pathway. Treatment with the ACE inhibitor, enalapril, counteracted UVB-induced wrinkles and skin damage (Matsuura-Hachiya et al. 2013).

Some studies have shown diverse effects of different representatives of ACE-Is on tissue collagen metabolism. Perindopril and ramipril prevented overexpression of type IV collagen mRNA in diabetic vessels (Cooper et al. 1998; Gilbert et al. 1998), while quinapril had no effect on collagen type I expression (Hernández-Presa et al. 1998).

Some studies demonstrated that captopril and enalapril improved pulmonary fibrosis in the lung tissue (Ghazi-Khansari et al. 2007). Early application of enalapril following dermal injury reduces formation of hypertrophic scars, probably because of its downregulatory effects on type III collagen production (Uzun et al. 2013). The mechanism of this process may involve TGF-β1 participation (Tang et al. 2009; Jiang et al. 2012; Uzun et al. 2013).

In fact, our data show that enalapril and enalaprilat both increase expression of TGF-β1. Moreover, other authors found that products of catalytic activity of prolidase, Pro and HyPro, induced increase in the amount of TGF-β1 and receptor expression for TGF-β1 (Surazynski et al. 2010).

Collagen is known as a ligand for α_2_β_1_ integrin. Previously, it has been shown that α_2_β_1_ integrin receptor is involved in signaling, which regulates collagen biosynthesis (Ivaska et al. 1999) and prolidase activity (Palka and Phang 1994, 1997). Another important point of collagen biosynthesis regulation is at the level of insulin-like growth factor-I receptor (IGF-IR). IGF-I is one of the most potent collagen-stimulating factor in collagen-synthesizing cells (Goldstein et al. 1989). Therefore, we considered α_2_β_1_ integrin and IGF-IR as a potential target in enalapril- and enalaprilat-induced increase of the above processes.

Our observations suggest that studied drugs increase collagen biosynthesis in fibroblasts primarily through increase of α_2_β_1_ integrin receptor and IGF-I-IR expressions. Presumably, increase in prolidase activity in fibroblasts due to enalapril and enalaprilat action is a result of increase of signaling by α_2_β_1_ integrin and IGF-IR. Both β_1_ integrin (Palka and Phang 1997) and IGF-IR (Miltyk et al. 1998) signaling were found to play important role in prolidase activity regulation.

Although several studies on animal models have shown that enalapril and enalaprilat treatment reduces fibrosis in some tissues, it cannot correspond to reduced collagen synthesis. Upregulation of collagen synthesis may reflect interstitial remodeling leading to increase or decrease of tissue collagen content depending on the rate of collagen degradation. It was supported by some studies showing that ACE inhibitors increase type III collagen synthesis (Claridge et al. 2004).

## Conclusion

The results of present study suggest that in fibroblasts, enalapril and enalaprilat may exert its effect on collagen biosynthesis through stimulation of prolidase activity and upregulation of expressions of prolidase, α_2_β_1_ integrin, IGF-IR, TGF-beta 1, and NF-κB p65.
